# Intersecting epidemics: deciphering the complexities of tuberculosis-diabetes comorbidity

**DOI:** 10.3389/ftubr.2024.1487793

**Published:** 2024-12-18

**Authors:** Mariana Araujo-Pereira, Caian L. Vinhaes, Beatriz Barreto-Duarte, Klauss Villalva-Serra, Artur T. L. Queiroz, Bruno B. Andrade

**Affiliations:** ^1^Laboratório de Pesquisa Clínica e Translacional (LPCT), Instituto Gonçalo Moniz, Fundação Oswaldo Cruz, Salvador, Bahia, Brazil; ^2^Multinational Organization Network Sponsoring Translational and Epidemiological Research (MONSTER) Initiative, Salvador, Bahia, Brazil; ^3^Instituto de Pesquisa Clínica e Translacional (IPCT), Faculdade Zarns, Clariens Educação, Salvador, Bahia, Brazil; ^4^Escola Bahiana de Medicina e Saúde Pública (EBMSP), Salvador, Bahia, Brazil; ^5^Departamento de Infectologia, Hospital das Clínicas da Faculdade de Medicina da Universidade de São Paulo, São Paulo, Brazil; ^6^Programa de Pós-Graduação em Clínica Médica, Universidade Federal do Rio de Janeiro, Rio de Janeiro, Brazil; ^7^Curso de Medicina, Universidade Salvador (UNIFACS), Salvador, Bahia, Brazil

**Keywords:** tuberculosis, diabetes mellitus, epidemiology, inflammation, multi-omics

## Abstract

Within the global health landscape, tuberculosis (TB) presents an ongoing challenge, demanding innovative strategies for its control. This review spotlights the intersection of TB with diabetes mellitus (DM), recognized by the World Health Organization as a key risk factor in the TB epidemic. Particularly prevalent in low and middle-income nations, the TB-DM comorbidity drives up TB rates through a nexus of chronic inflammation. By delving into the epidemiological, clinical, and inflammatory dimensions, we elucidate the impact of TB-DM on patient prognosis and the multifaceted complications it introduces to disease transmission, diagnosis, and treatment protocols. Our synthesis aims to offer a fresh lens on TB-DM, fostering a nuanced understanding that could inform future healthcare policies and interventions.

## 1 Introduction

In the contemporary global health landscape, tuberculosis (TB), a persistent challenge, intersects intricately with another widespread condition, diabetes mellitus (DM) (1, 2). Recognized by the World Health Organization (WHO) as a crucial risk factor in the TB epidemic, TB-DM comorbidity emerges as a significant concern, especially in low and middle-income countries (1). The coexistence of TB-DM presents a unique challenge in global health, demanding a nuanced understanding of their interplay, that affects TB disease progression and individual outcomes. An integrated approach, incorporating both traditional epidemiological methods and advanced molecular techniques, is essential to fully comprehend and effectively address the TB-DM comorbidity.

TB remains one of the major causes of death by a single pathogen worldwide, leading to a global health concern, with the disease affecting around 10 million people each year (1). The interplay between the *Mycobacterium tuberculosis (*Mtb) and the host, mediated by the inflammatory responses, determines a wide spectrum of clinical presentations. The challenge of reducing the disease burden arises from different factors, including the influence of different comorbidities, mainly those that affect the quality of inflammatory responses, such as HIV (3), malnutrition (4) and DM.

DM is a chronic metabolic disorder that represents a dramatically high burden to healthcare systems worldwide. It has been reported that around half a billion people live with this disease (2), mainly in low- and middle-income countries. Additionally, DM is a significant contributor to global morbidity and mortality, leading to an array of severe complications, including kidney failure, stroke, and heart disease, and is directly responsible for a substantial number of deaths annually (2). DM affects the metabolism through multiple mechanisms, many of them related to the activation of poorly controlled pro-inflammatory pathways (5, 6), that influence disease progression and susceptibility to infectious diseases, such as COVID-19 (7) and TB (8).

In this context, the TB-DM comorbidity has garnered significant attention in the last years. Interestingly, some studies revealed regional disparities in the immune profile resulting from this interaction and emphasized the influence of socio-demographic and clinical factors on both diseases (9, 10). This article delves into the multifaceted relationship between TB and DM, exploring how this interplay exacerbates TB incidence. Our review compiles recent findings on the epidemiological, clinical, and inflammatory aspects of this nexus, highlighting its profound implications on patient outcomes and the broader challenges it poses in disease management. By scrutinizing the TB-DM interconnection, we aim to provide a comprehensive multiplatform perspective that not only sheds light on the complexities of this comorbidity but also suggests pathways for innovative healthcare strategies and policy formulations.

References for this review were identified through searches of PubMed for articles published from January, 1924, to January, 2024, by use of the terms “tuberculosis,” “diabetes,” “hyperglycemia,” “epidemiology,” “immune profile,” “treatment outcomes,” “clinical presentation,” and “omics.” Articles resulting from these searches and relevant references cited in those articles were reviewed, and the most relevant and recent papers published in English were included.

## 2 Epidemiological trends of DM among TB patients

The intersection of DM and TB poses a global health challenge, as DM is recognized by the WHO as a primary risk factor for new TB cases. Interestingly, many of the top 10 countries with the highest DM rates are also significant contributors to the global TB burden. Additionally, in these low and middle-income countries the prevalence of DM is increasing, creating a dual challenge that has serious public health implications (11). In a modeling study, conducted in 13 high endemic TB countries, the authors used dynamic tuberculosis transmission models to examine the impact of DM on TB epidemiology (12). The previous prevalence of DM in each of the 13 countries was accessed to simulate the future DM prevalence and a country-specific model calibrated to estimate the trend of TB incidence (12). Results show that lowering or stopping the rise of DM can avoid 6 million TB incident cases and 1.1 million deaths due to TB in the 13 countries for 20 years (12). Therefore, understand the epidemiological and clinical patterns of DM in TB patients is vital for delivering patient-centered care and managing these diseases to reduce the global TB impact (1).

The global prevalence of DM in TB cases exceeds 15%, surpassing the prevalence in the general adult population by over 50% in 2021 (13, 14). A recent metanalysis, including 2.3 million TB patients, identified that this trend is notably higher in North America (19.7%), Western Pacific (19.4%), Southeast Asia (19.0%), and Middle East and North Africa (17.5%) (13). While these variations in DM prevalence among TB patients appear to correlate with the general DM prevalence in each region, disparities within the same regions suggest more intricate underlying factors. Of note, these factors could be associated with the clinical management of the patients, that could vary according to the region. India and Sri Lanka, for example, possess a significantly higher burden of TB-DM than the other countries in the South Asia region (13, 15). This may be a reflection of the high coverage of DM screening in TB clinics, as well as points to the potential influence of regional epidemiological patterns, consumption habits, environmental factors, and genetic predispositions, now being explored through multiplatform studies, indicating a complex interplay of epidemiological elements in TB-DM comorbidity.

Despite well established relationships between the diseases, it is important to note that TB, as an infectious disease, could lead to hyperglycemia. Therefore, the use of screening algorithms for transient hyperglycemia displays pivotal role. In a case-control study conducted in Tanzania between July 2012 and June 2014, TB patients before treatment were paired with volunteers by sex and age. All participants underwent DM tests, as fasting capillary glucose, 2-h capillary glucose and HbA1c levels at enrollment (16). TB patients were submitted again to the tests after the therapy, and the authors found a significant decrease in DM prevalence (16), highlighting the relevance of DM confirmation after TB treatment to avoid an overestimation of the dual burden of diseases.

The prevention of progression from infection to disease is crucial to control the TB diseases burden worldwide (1). Currently, the identification of people infected with *Mtb* can be performed with tuberculin skin test (TST) or interferon-γ release assay (IGRA). Both tests are based on the host immune response against the bacilli, and could be affected by secondary morbidities that affect the inflammatory responses, such as DM. Some reports have associated DM with increases in the indeterminate IGRA results (17). However, the association has been controversial in other studies, where DM was not associated with indeterminate results (18). A Brazilian study performed with 553 participants from a high endemic area, highlight the QuantiFERON-TB Gold as a good tool to screening TB infection in DM (19), a susceptible population to TB infection and diseases. Of note, data suggests also a higher incidence of DM among those with TB infection (20). A retrospective cohort performed in the USA among US Veterans included patients who received TST or IGRA during 2000 to 2015. Patients with previous DM diagnosis were excluded, and the participants followed from TST/IGRA test date until date of DM diagnosis, death or 2015 (20). The results revealed a higher DM incidence among LTBI, when compared with those without positive TST/IGRA, 1.012 vs. 744; HR 1.4 [95% CI 1.3–1.4] (20). Therefore, the investigation of TB infection in DM patients, as well as dysglycemia in TB infected people, is crucial as a cost-effective public health strategy to mitigate the impact of the epidemics.

The epidemiological factors associated with the TB-DM dynamicity have been the focus of several studies. Among the known risk factors, advanced age was consistently identified as a prominent risk factor for TB-DM comorbidity (21, 22). Lifestyle habits, such as sedentarism (23, 24) behavior, along with socio-demographic factors like urban living and high-income status (25, 26) have also been recognized as elements that increase the TB-DM prevalence. Clinical variables such as a family history of DM (24, 27, 28) further, contribute to an increase in the risk of TB-DM. However, the relationship between Body Mass Index (BMI) and TB-DM is unclear, with both low and high BMI values, as well as obesity and malnutrition, having been separately associated with TB-DM (24, 29). Furthermore, genetic factors at the population level may significantly influence the occurrence of TB-DM comorbidity. A study on Indian TB-DM individuals and their household contacts (HHC) observed that HHCs who developed TB, had a specific genetic pattern called the “GG genotype” of the interleukin(IL)-6−174G>C gene, indicating a genetic component in TB susceptibility (30). IL-6 is a cytokine essential for initiating and regulating the immune response to Mtb. Genetic variants affecting IL-6 can influence the immune system's ability to combat TB, impacting who may develop the disease after exposure (30). The insights from epidemiological and molecular studies on TB-DM comorbidity have significant implications for future healthcare policies and interventions. The relationship between clinical, lifestyle and genetic factors, such as the IL-6 GG encourage the design of molecular epidemiology projects to identify specific SNPs associated with increased disease risk, explain varied disease burdens across populations and inform tailored public health strategies, potentially reducing the incidence and improving the management of TB-DM comorbidity.

## 3 Dissecting the clinical interplay and implications of TB-DM comorbidity

The relationship between TB-DM represents a complex, bidirectional nexus significantly impacting clinical presentation, and disease dynamics and outcomes. DM is not only associated with the prevalence of TB but also exacerbates its progression (31, 32). By 2050, projections suggest that one-third of TB incidence and mortality within the Asia-Pacific region and similar environments, will be attributable to DM (33). This alarming trend underscores the need for integrated health strategies that address both TB and DM, particularly in regions with high prevalence rates.

Clinically, DM complicates TB management. In recent research spearheaded by our team, we uncovered a positive association between DM and increased mycobacterial loads, as well heightened Acid-Fast Bacilli (AFB) positivity, at diagnosis in TB-DM participants if compared with those without DM (32, 34). Furthermore, DM has been implicated in the presence of distinct lung lesions in chest radiographs of TB-DM patients in our Brazilian cohort (35). The presence of DM also influences the clinical presentation of TB, increasing the occurrence of TB symptoms such as hemoptysis, night sweats, and weight loss, as well as elevates the clinical severity (34–38). In contrast, TB-DM patients exhibited lower fever, reduced cough, and less sputum production, leading to delays in diagnosis and initiating appropriate treatment (39). The changes in clinical presentations and the consequences of a late diagnosis and start of TB treatment emerge as a clinical challenge in TB management and disease burden control.

In addition, the available evidence suggests that the prevalence of DM is higher among Drug Resistant (DR)-TB and Multidrug Resistant (MDR)-TB patients compared to the general population. A meta-analysis conducted in 2017 with 13 studies and 9,289 individuals shown a significant association between DM and MDR-TB (OR 1.7 [95% CI = 1.32–2.22]) (40). A more recent meta-analysis performed with 30 studies and including 225.812 patients also demonstrated a risk of MDR-TB among DM (HR 0.81 [95% CI: 0.60–0.96]) (41).

It is important to emphasize that DM also impacts anti-TB treatment, being associated with unfavorable outcomes, such as death, treatment failure and relapse cases (15, 42). Specifically, mortality and anti-TB treatment failure have been consistently linked with DM and higher glycated hemoglobin (HbA1c) levels (15, 43–46). Of note, treatment failure is more pronounced in TB-DM comorbidity within low- and middle-income countries (47), where DM conferred a 3.9 times increased risk of treatment failure in contrast with TB-only patients (48, 49). A pooled metanalysis also showed that TB relapse risk increases in TB-DM individuals (15, 50). Furthermore, DM was associated with poor outcomes in DTR-TB, with a risk of 1.56 times increased risk for unsuccessful outcomes (51). Following treatment completion, patients with TB-DM frequently require more extensive long-term care due to their challenges with managing blood sugar levels, elevated risk of cardiovascular disease, and potential for kidney damage (1).

DM also affects another important nexus to TB control: the transmission cascade. The key factors are the increased bacterial load, delayed diagnosis and unfavorable treatment outcomes in TB-DM patients, as abovementioned, which directly contributes to a higher transmission of Mtb. In a study from our group, conducted with a longitudinal multicenter cohort, it was observed that TB-DM patients shown an increased risk of Mtb transmission to their close contacts, if compared to TB-only patients (52). These factors interact in ways that enhance the transmission potential among TB-DM patients, thereby underscoring the need for timely diagnosis and targeted interventions to control the spread of TB in this vulnerable population.

On the other hand, antitubercular therapy also affects DM management. Due to drug interaction with rifampicin, a pivotal drug in TB treatment, and changes in food consumption and metabolic demand, with decreasing in systemic inflammation and consequently increasing in appetite, the targeted HbA1c level could be difficult to achieve in TB-DM (53). Additionally, despite metformin is not metabolized by P450 enzymes (53), the drug is a substrate for human transporters. Rifampicin could affect the expression of organic cation transporter (OCT1) leading to hepatic uptake of metformin, that could interfere in glucose levels in health participants (54). However, a pharmacodynamic study in TB-DM patients showed that rifampicin leads to changes in plasmatic concentration of metformin, but do not lead to changes in glucose levels (55). Side effects in metformin use with rifampicin include gastrointestinal effects and rarely lactic acidosis (56). These effects could contribute to poor adhesion of both treatments, leading to a cascade of complications, highlighting the relevance of TB-DM to public health.

The challenges of TB-DM regarding clinical manifestations and, consequently, outcomes play pivotal role in the future direction of TB management. First, is necessary to expand the glycemic tests among TB patients, identifying and treating DM with better glucose control. On the other hand, among those DM patients, an active search for TB is fundamental to early diagnosis and treatment. In addition, it is important to focus on screening close contacts of TB-DM individuals, given the higher risk of TB infection. Finally, improving the understanding of the molecular mechanisms associated with the worse clinical presentation and unfavorable outcomes could help to tailoring effective and patient-centered interventions, improving treatment, and contributing with the burden control.

## 4 Cellular and molecular mechanisms underlying TB-DM comorbidity

The varying global incidences of TB-DM and the notable impact of DM on the clinical presentation, outcomes, and transmission of TB underscore a complex and intricate synergy between these conditions. In this landscape, multi-platform approaches are essential for dissecting the intricate cellular and molecular interactions in TB-DM comorbidity and provide a comprehensive view of the inflammatory processes involved. Such advancements have the potential to significantly enhance prognosis, follow-up, and contribute to a reduction in the burden of TB. In this context, several multimolecular biomarkers have been explored with the goal of enhancing diagnosis and clinical management of TB-DM patients.

### 4.1 Cellular immunology aspects

It becomes evident that the interplay between TB and DM significantly alters immune cell function and response. However, the mechanisms by which immune responses are impaired in individuals with TBM-DM are not fully understood, being complex and multifactorial. In TB infection, the effector functions of alveolar macrophages are crucial to containing the infection within the lungs (57). However, in DM patients, the functionality of these cells is decreased due to metabolic alterations associated with hyperglycemia (58, 59). DM impairs the functional activity of neutrophils (60) and reduces macrophage migration to sites of infection (57). Animal studies with diabetic mice have shown a delay in innate immune response initiation, which includes a compromise of nitric oxide production and phagocytic cell functionality, notwithstanding cytokine stimulation (57). In this same model, alveolar macrophages exhibited increased expression of CCR2, which potentially hampers the migration of monocytes to the lungs. Therefore, this may result in a compromised capability to kill intracellular Mtb, thus further contributing to both infection susceptibility and increased bacterial load (57, 61, 62). In addition, high blood glucose levels negatively impact the antigen presentation capabilities, which are vital for initiating adaptive immune responses against Mtb (63).

Similarly, the T cell response is notably affected, with DM patients often displaying dysregulated T cell responses. This dysregulation is hallmarked by imbalance in T helper (Th) cell subsets, with decreased Th1 responses and increased Th2 and Th17 responses. Such an imbalance can significantly alter the host ability to mount an effective response against TB. In a study comparing euglycemic and diabetic mice, it was observed that at the onset of infection diabetic mice exhibited a delayed activation of the adaptive immune system. This delay was indicated by decreased production of IFN-γ and fewer Mtb antigen (ESAT-6) presence compared to euglycemic mice (64, 65). Studies have shown that the frequencies of T cells producing type 1 and type 17 cytokines are significantly reduced in TB-DM patients compared to those without DM. This suggests a compromised ability to mount an effective immune response against Mtb due to altered cytokine signaling (66).

The interplay between TB-DM leads to significant impairments in both innate and adaptive immune responses. This results from a combination of altered cytokine production, diminished T cell functionality, innate immune cell dysfunction, phenotypic changes in immune cell populations, and metabolic influences due to hyperglycemia. These cellular alterations contribute to a weakened immune defense against TB in diabetic individuals, underlining the importance of targeted interventions that address these specific cellular immune challenges in TB-DM comorbidity.

### 4.2 Genomics

Multi-omics research has delved deeper into the layers of complexity in TB-DM comorbidity. Genomics, proteomics, and transcriptomics, each provide unique insights into the pathophysiological mechanisms in TB-DM. Genomic studies, for instance, have identified genetic variants that predispose individuals to TB-DM, revealing potential targets for personalized medicine approaches. Polymorphisms on IL-6 and IL-18 genes were associated with TB-DM comorbidity and the occurrence of TB in close contacts (30). Another study analyzed the interferon-gamma gene variants and found that the TACCCAGA haplotype was negatively associated with TB-DM. The frequency of this haplotype was high in healthy controls compared to TB-DM patients, ehich may denote the importance of genetic variation in TB-DM predisposition, as well as facilitate the identification of individuals at risk.

Currently, new approaches have been applied to better understand genetic variations and predispositions to TB in DM population. Using Mendelian randomization, a recent study selected 152 independent single-nucleotide polymorphisms (SNPs) as instrumental variables to evaluate genetic causality between type 2 DM and TB (67). Results reveal an increased risk of PTB among type 2 DM in the East Asian population (67). Evaluating the causal relationship between type 1 DM and TB, a Chinese group assessed SNPs of type 1 DM and PTB (68). Additionally, data from Genome-Wide Association Study (GWAS) were utilized to explore clinical traits of DM, such as glycemic traits, lipids and obesity (68). Using inverse variance weighting method (IVW), weighted median method, and Mendelian randomization-Egger regression were used to evaluate the causal relationship, the group identified that type 1 DM and HDL-C were risk factors to PTB (68).

These findings collectively deepen our understanding of the genetic interplay between TB and DM, emphasizing the need for integrative approaches that consider genetic, metabolic, and environmental factors in addressing TB-DM comorbidity.

### 4.3 Transcriptomics

In the realm of transcriptomics, investigations in samples from TB-DM patients have illuminated the molecular pathways that may be dysregulated in this comorbidity. The superposition of TB-DM is marked by chronic inflammation, alongside qualitative and quantitative changes in immune activation characterized by distinct gene expression patterns. In a recent multi-center cohort study involving TB-DM individuals (69), pathway enrichment analysis had shown a notable trend toward heightened neutrophil and innate immune pathway activation in TB-DM participants even after anti-TB treatment commencement (69), that might reflect persistent inflammation. The findings of this same study unveiled that the genetic and immune responses may vary across different geographical regions (69), as discussed in the sections above. Additionally, the study dissect the correlations between the HbA1c levels and some biological pathways, highlighting positive correlation between HbA1c levels and pathways associated with insulin resistance, metabolic dysfunction, diabetic complications, and chromosomal instability (69). These correlations may play a pivotal role in the pathophysiology of TB-DM, contributing to a more severe clinical presentation and unfavorable outcomes.

Another multicentric study has found that DM amplifies the expression levels of genes related to the innate inflammatory response and reduces genes related to the adaptative immune response in TB individuals (70). A decreased type I interferon (IFN) response was identified in TB-DM participants if compared to TB-only patients. Despite IFN-γ be the most important IFN type against *Mtb*, with direct effect in macrophage activation, previous studies have identified an up-regulation in type I IFN in TB only patients when compared to controls (71–74). Therefore, type I IFN has also been associated with TB pathophysiology (74, 75). Although the excessive IFN responses in TB only has been associated with a deleterious activity (70). Additionally, a Chinese study revealed 952 differentially expressed genes (DEGs) in TB-DM, enriched in pathways associated with the cell cycle, homeostasis, and immunological processes, highlighting changes in several biological pathways induced by DM in TB patients (76). Expanding the scope of transcriptomic studies in TB-DM in a Brazilian cohort, the long non coding (lnc)RNA expression analysis led to the identification of a distinct lncRNA signature, which effectively distinguishes TB-DM from TB-only cases with an accuracy of 90%−94%. Notably, the lncRNAs included in the signature (LINCO2009, LINCO2471, ADM-DT, and GK-AS1) hold a critical role in the pathways related to inflammatory activation against Mtb (77). These studies demonstrate that transcriptomics has shed light on the field of TB-DM, revealing that there are consistently altered pathways in TB-DM patients.

### 4.4 Proteomics

Proteomic analyses, through the quantification of cytokines, chemokines, and other immune-related proteins, have significantly advanced in the understanding of immune responses in TB-DM. This approach has been essential in revealing how hyperglycemia-induced metabolic alterations in DM patients impair the functionality of the innate and adaptive immune responses in TB-DM. Proteomic data in TB-DM also has the potential to reveal key alterations in protein expression and point toward potential novel biomarkers. These insights are crucial in delineating the complex dynamics of immune dysfunction in TB susceptibility, transmission, and treatment outcomes (57, 64, 65, 78–81).

In TB-DM patients, proteomic data indicates an increase in proteins involved in the complement and coagulation cascade, as well as in cholesterol metabolism. This elevation suggests a potential link between lipid metabolism dysregulation and the heightened inflammatory state observed in TB-DM comorbidity (82). In another study, 18 differentially expressed protein spots were identified in TB-DM patients. These alterations were associated with potential metabolic complications specific to TB-DM and shifts in proteins governing cell cycle and growth regulation hint at disrupted processes like cell proliferation and apoptosis (83).

A recent mice study evaluates the changes induced by TBDM in tissue level. Specifically in liver, a set composed of 60 proteins shows deregulation in TBDM when compared to TB only mice. These proteins have been associated with small molecule catabolic process, retinol metabolism, polyol biosynthetic process, cysteine, and methionine metabolism (84). Functional analysis reveals perturbations in 20 functional modules, that include monocarboxylic acid metabolism, amino acids biosynthesis, cysteine and methionine metabolism, retinol metabolism, monocarboxylic acid metabolic process, polyol biosynthetic process, steroid hormone biosynthesis, small molecule catabolic, and biosynthetic process (84). Of note, cysteine and methionine are associated with glutathione metabolism, and consequently contributing with the balance of the oxidative stress. Is know that the imbalance in reactive oxygen species contributing with tissue damage in TB (85). Therefore, the study reveals that the alterations in livre proteomic induced in TBDM leads to progression of liver diseases (84). These findings not only provide a deeper understanding of TB-DM pathophysiology but also open avenues for new diagnostic, monitoring, and treatment strategies.

DM patients also present an altered cytokine milieu that favors immune dysregulation. In several studies, with TB and TB-DM individuals, it was observed that DM participants experienced higher levels of inflammatory activation than those without DM (81, 86). Patients with TB-DM have higher levels of pro-inflammatory cytokines such as IFN-γ, IL-1β, and IL-17, as well as lower levels of anti-inflammatory cytokines such as IL-10, compared to patients without DM. Additionally, throughout the anti-TB treatment, these markers remained elevated for a longer period in TB-DM patients compared to non-DM individuals, which characterized persistent hyperinflammation in this group of individuals (86). In summary, the comorbidity of TB-DM is hallmarked by chronic and unbalanced inflammation, reflected in abnormal levels of proteins, and the superposition of these disorders leads to qualitative and quantitative changes in immune activation (69).

### 4.5 Lipidomic

Lipidomic changes have been studied in the context of TB-DM, and it is known that the association TBDM leads to an altered lipidomic (87). Some studies suggest that these altered lipids levels are associated with the increased TB risk in DM patients. In this context, glycerophospholipids were studied (88). The study identified 14 glycerophospholipids differentially regulated between TB and TB-DM, emerging as potentially biomarkers in the field (88). Using lipidomic approaches to identify persistent hyperinflammation by evaluating urinary lipid mediator profiles of participants with TB and TB-dysglycemia, it was observed that levels of a urinary metabolite of prostaglandin 2 (PGE-M) and leukotriene 4 (LTE4) were consistently higher during anti-TB treatment in the DM group compared to the normoglycemic group. These lipid mediators play a crucial role in modulating the immune response (89). Interestingly, in an adjusted multivariable model TB-DM was independently associated with increased concentrations of PGD-M, PGI-M, and LTE4 at baseline (89). This profile of higher metabolites expression in TB-DM patients if compared to TB-only patients helps explain why these individuals present severe symptoms and more enduring lung damage more often (86), which can be associated with unfavorable outcomes and Mtb dissemination.

### 4.6 Metabolomics

Metabolomics, by analyzing the small molecule metabolites present in TB-DM patients, offers insights into the metabolic disruptions caused by the interplay of TB and DM. In studies exploring metabolomics, specific metabolic changes induced by TB-DM were delineated (90). Plasma amine and acylcarnitine levels were measured in TB and TB-DM patients, with partial least squares discrimination analysis showing robust group discrimination (90). Notably, TB-DM exhibited lower levels of choline, glycine, serine, threonine, and homoserine compared to TB-only patients. Of note, the levels of these metabolites did not normalize during treatment (90). In a recent Korean study, plasma metabolic profiles of TB and TB-DM were investigated using metabolomics and lipidomics (87). TB-DM participants presented higher concentrations of bile acids and molecules related to carbohydrate metabolism, as well as the depletion of glutamine, retinol, lysophosphatidylcholine, and phosphatidylcholine (87). Arachidonic acid metabolism, crucial for eicosanoid production, emerged as a key factor in TB-DM pathophysiology (87). Eicosanoids, extensively studied in TB and TB-DM (89, 91), emerges as potential markers for diseases severity (80).

### 4.7 Integrative analysis

Multi-omics investigations have significantly advanced our comprehension of the complex interplay between these two diseases. By integrating data from various omics layers such as genomics, transcriptomics, proteomics, metabolomics, and epigenomics, it is possible to achieve a holistic understanding of the biological processes involved in TB-DM interaction and consequently prognosis. Funding multi-omic studies is fundamental to better understanding the pathophysiology of TB-DM and its impact on anti-TB treatment outcomes as well as in the identification of new targets to host directed therapies.

Using whole blood gene expression and plasma analytes (81), a groundbreaking study identified that DM in comorbidity with TB intensifies the neutrophilic inflammatory response, possibly indicative of a higher bacterial load or a distinct disruption in immune function. This heightened response was marked by increased plasma levels of cytokines and growth factors, as well as differentially expressed genes, that differentiate individuals with TB-DM from the majority of those with only TB or DM (81). Intriguingly, the expression patterns of TB-DM-exclusive genes were linked to critical biological processes and therapeutic targets. They were associated with endoplasmic reticulum stress, a vital cell stress response, and showed connections to the mechanisms of action of the antibiotic doxycycline and anti-cancer drugs such as 5-fluorouracil and semaxanib (81).

In another study, using a multi-platform approach to integrate clinical, transcriptomic, lipidomic, and proteomic data from a Brazilian TB-DM cohort, were identified several multimolecular baseline markers—MMP-28, LTE-4, 11-dTxB2, PGDM, FBXO6, SECTM1, and LINCO2009—that effectively differentiate between TB-DM, TB-only, DM-only, and healthy control groups (92). After anti-TB treatment onset, a notable decrease in these markers was observed, correlating with microbiological cure. Significantly, markers such as 11-dTxB2, SECTM1, and LINCO2009 not only emerged as indicators for new host-directed TB treatments but also as potential predictors of treatment outcomes (92). Furthermore, this integrated molecular signature demonstrated high accuracy in distinguishing TB-DM cases from TB-only, DM-only, and healthy control (without TB and DM) groups in Brazil and was validated in three external cohorts, outperforming signatures derived solely from transcriptomic data (69, 92). Crucially, these findings highlight that multimolecular signatures can be more predictive and impactful for precision medicine compared to single-omic approaches, underscoring the enhanced potential of multi-omic platforms in advancing our understanding of inflammatory and infectious diseases, as well in finding markers that can be implemented in the clinical practice.

## 5 Paving the path for future breakthroughs

The studies included in this review provide substantial evidence of the interplay between TB and DM and highlight the need for advanced research methodologies. Current evidence in epidemiology demonstrates a global prevalence of DM in TB cases, emphasizing age, lifestyle, socio-economic factors, family history, and hypertension as key risk factors. However, there exists a knowledge gap that needs addressing to understand the regional disparities in TB-DM comorbidity. Investment in molecular epidemiology studies is crucial for this understanding and is pivotal for developing targeted public health strategies. This approach would not only elucidate regional differences but also aid in formulating more effective, region-specific interventions.

The clinical nexus of TB-DM presents a bidirectional impact, with DM complicating TB management and exacerbating disease progression. Research shows a positive association between DM and increased mycobacterial loads and distinct lung lesions, underscoring the need for integrated health strategies addressing both diseases. The next step in addressing TB-DM comorbidity in the clinical point of view would involve developing more targeted public health policies for individuals with both conditions. This could include enhanced TB screening in DM patients and the other way around, as well as expanding research into contacts of these patients to assess transmission dynamics. It is also important to highlight that further studies are needed to evaluate how DM multimorbidity (such as chronic kidney disease, fatty liver, and cardiovascular problems) affect the inflammatory profile of the TB-DM patients, making complex the potential identification of biomarkers or treatment targets. These strategies would improve individual patient care and contribute to broader public health efforts in managing and preventing the spread of TB-DM comorbidity.

It is also known that DM impacts immune cell function and response in TB, with specific genetic variations associated with TB susceptibility. This points to the potential of using advanced technologies like single-cell analysis to uncover new therapeutic targets and biomarkers. This review provides several cellular and molecular insights associated with TB-DM comorbidity. We discussed the altered immune cell function in DM patients which are crucial in containing TB infection, as well as the influence of genetic factors, such as polymorphisms, and the role of multi-omics in understanding molecular pathways disrupted in TB-DM. Building on this, the use of multi-platforms, as well as the addition cutting-edge technologies such as single-cell analysis could be instrumental. This technology can allow for a more granular understanding of cellular responses in TB-DM comorbidity at an individual cell level, potentially uncovering new pathways and therapeutic targets. In addition, the development, validation, and implementation of point-of-care testing for specific biomarkers already identified through these advanced methods could revolutionize early detection and monitoring of TB-DM comorbidity. This approach aligns with the development of predictive scores, integrating genetic, molecular, and clinical data to accurately assess disease progression and treatment outcomes. Additionally, considering the immune dis-function, a targeted TB vaccine could play crucial role in diseases prevention. Some TB vaccines has been developed, but anyone directed to population with impaired inflammatory responses. A better understanding of the nuances of immune activation and impairment in TB-DM could help the development of a new TB vaccine focused on DM patients.

**Figure 1:**
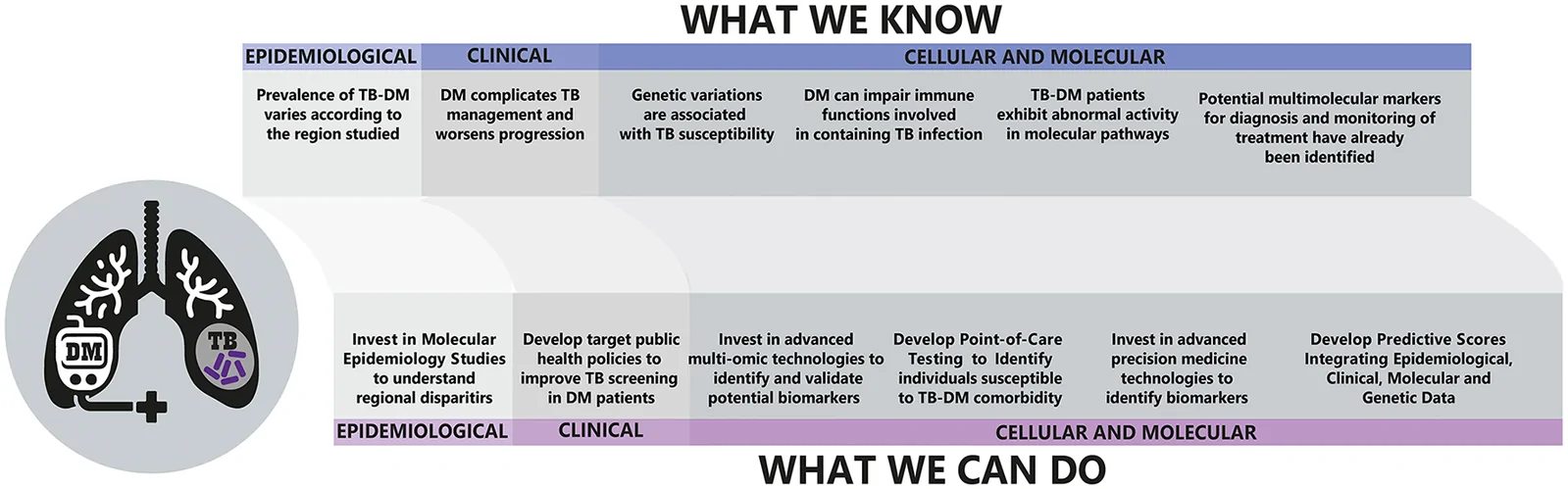
Overview of the interplay between Tuberculosis (TB) and Diabetes Mellitus (DM) and future directions. **Upper**: Current epidemiological, clinical, and cellular/molecular knowledge concerning TB-DM co-morbidity. **Down**: Prospective actions for advancing research and healthcare strategies.

Moreover, the creation and improvement of comprehensive risk scores, incorporating socio-demographic, lifestyle, and clinical variables, could greatly enhance the precision of public health interventions. These scores, derived from multi-omic and epidemiological data, could be tailored to specific populations, considering regional variations in TB-DM comorbidity. Figure 1 encapsulates the current state of knowledge and future directions in TB-DM comorbidity research. In essence, leveraging these innovative technologies and approaches could bridge the gap between current knowledge and the untapped potential in managing TB-DM comorbidity, leading to more effective, personalized treatment, and prevention strategies.

The intricate relationship between TB-DM is a worldwide health threat, impacting treatment outcomes and mortality rates. The synthesis of epidemiological, clinical, genomic, transcriptomic, proteomic, and lipidomic studies is vital for understanding the complexities of TB-DM comorbidity. The study of multi-omic platforms emerges as an opportunity to gain insights into disease pathogenesis, given that it simultaneously explores several components of immune responses through multiple assay platforms. The identification of precise biomarkers for diagnosis and individualized treatment, along with public health strategies informed by molecular and epidemiological findings, is crucial. This area of research holds the promise of significant advancements, offering enhanced management of TB-DM comorbidity and contributing to global public health outcomes.

## References

[1] W.H. Organization. Global Tuberculosis Report. Geneva (2023).

[2] W.H. Organization. Global report on diabetes. Geneva (2016).

[3] Bell LCK Noursadeghi M. Pathogenesis of HIV-1 and *Mycobacterium tuberculosis* co-infection. Nat Rev Microbiol. (2018) 16:80–90. 10.1038/nrmicro.2017.128 29109555

[4] Gupta KB Gupta R Atreja A Verma M Vishvkarma S. Tuberculosis and nutrition. Lung India. (2009) 26:9–16. 10.4103/0970-2113.45198 20165588 PMC2813110

[5] Tsalamandris S Antonopoulos AS Oikonomou E Papamikroulis GA Vogiatzi G Papaioannou S. The role of inflammation in diabetes: current concepts and future perspectives. Eur Cardiol. (2019) 14:50–9. 10.15420/ecr.2018.33.1 31131037 PMC6523054

[6] Berbudi A Rahmadika N Tjahjadi AI Ruslami R. Type 2 diabetes and its impact on the immune system. Curr Diabetes Rev. (2020) 16:442–9. 10.2174/1573399815666191024085838 31657690 PMC7475801

[7] Roy B Runa SA. SARS-CoV-2 infection and diabetes: pathophysiological mechanism of multi-system organ failure. World J Virol. (2022) 11:252–74. 10.5501/wjv.v11.i5.252 36188734 PMC9523319

[8] Restrepo BI. Diabetes and tuberculosis. Microbiol Spectr. (2016) 4:1–21. 10.1128/microbiolspec.TNMI7-0023-2016 28084206 PMC5240796

[9] Al-Rifai RH Pearson F Critchley JA Abu-Raddad LJ. Association between diabetes mellitus and active tuberculosis: a systematic review and meta-analysis. PLoS ONE. (2017) 12:e0187967. 10.1371/journal.pone.0187967 29161276 PMC5697825

[10] Foe-Essomba JR Kenmoe S Tchatchouang S Ebogo-Belobo JT Mbaga DS Kengne-Nde C. Diabetes mellitus and tuberculosis, a systematic review and meta-analysis with sensitivity analysis for studies comparable for confounders. PLoS One. (2021) 16:e0261246. 10.1371/journal.pone.0261246 34890419 PMC8664214

[11] Restrepo BI Fisher-Hoch SP Crespo JG Whitney E Perez A Smith B. Type 2 diabetes and tuberculosis in a dynamic bi-national border population. Epidemiol Infect. (2007) 135:483–91. 10.1017/S0950268806006935 16863600 PMC2870584

[12] Pan SC Ku CC Kao D Ezzati M Fang CT Lin HH. Effect of diabetes on tuberculosis control in 13 countries with high tuberculosis: a modelling study. Lancet Diabetes Endocrinol. (2015) 3:323–30. 10.1016/S2213-8587(15)00042-X 25754415

[13] Noubiap JJ Nansseu JR Nyaga UF Nkeck JR Endomba FT Kaze AD. Global prevalence of diabetes in active tuberculosis: a systematic review and meta-analysis of data from 2.3 million patients with tuberculosis. Lancet Glob Health. (2019) 7:448–460. 10.1016/S2214-109X(18)30487-X 30819531

[14] Workneh MH Bjune GA Yimer SA. Prevalence and associated factors of tuberculosis and diabetes mellitus comorbidity: a systematic review. PLoS ONE. (2017) 12:e0175925. 10.1371/journal.pone.0175925 28430796 PMC5400500

[15] Gautam S Shrestha N Mahato S Nguyen TPA Mishra SR Berg-Beckhoff G. Diabetes among tuberculosis patients and its impact on tuberculosis treatment in South Asia: a systematic review and meta-analysis. Sci Rep. (2021) 11:2113. 10.1038/s41598-021-81057-2 33483542 PMC7822911

[16] Boillat-Blanco N Ramaiya KL Mganga M Minja LT Bovet P Schindler C. Transient hyperglycemia in patients with tuberculosis in tanzania: implications for diabetes screening algorithms. J Infect Dis. (2016) 213:1163–72. 10.1093/infdis/jiv568 26609005

[17] Chee CB Gan SH Khinmar KW Barkham TM Koh CK Liang S. Comparison of sensitivities of two commercial gamma interferon release assays for pulmonary tuberculosis. J Clin Microbiol. (2008) 46:1935–40. 10.1128/JCM.02403-07 18400912 PMC2446854

[18] Shin HJ Kim TO Oh HJ Park HY Chang JS Ahn S. Impact of diabetes mellitus on indeterminate results of the QuantiFERON TB Gold In-Tube test: a propensity score matching analysis. PLoS ONE. (2017) 12:e0181887. 10.1371/journal.pone.0181887 28732078 PMC5521843

[19] Torres AV Corrêa RD Bevilacqua MD do Prado LC Bandeira FM Rodrigues LS. Screening of latent tuberculosis infection among patients with diabetes mellitus from a high-burden area in Brazil. Front Clin Diabetes Health. (2022) 3:914574. 10.3389/fcdhc.2022.914574 36992754 PMC10012069

[20] Magee MJ Khakharia A Gandhi NR Day CL Kornfeld H Rhee MK. Increased risk of incident diabetes among individuals with latent tuberculosis infection. Diabetes Care. (2022) 45:880–7. 10.2337/dc21-1687 35168250 PMC9016736

[21] Mabula PL Kazinyingia KI Chavala EC Mosha V Msuya SE Leyaro BJ. Prevalence and risk factors for diabetes mellitus among tuberculosis patients in Moshi Municipal Council, Kilimanjaro Tanzania. East Afr Health Res J. (2021) 5:69–74. 10.24248/eahrj.v5i1.658 34308247 PMC8291211

[22] Alturki S Al Amad M Mahyoub E Al Hanash N Alhammadi A. Prevalence of diabetes mellitus among patients with tuberculosis and its associated factors in Sana'a, Yemen, 2021. Epidemiologia. (2023) 4:202–11. 10.3390/epidemiologia4020021 37367186 PMC10296863

[23] Balde NM Camara A Camara LM Diallo MM Kake A Bah-Sow OY. Associated tuberculosis and diabetes in Conakry, Guinea: prevalence and clinical characteristics. Int J Tuberc Lung Dis. (2006) 10:1036–40. 16964797

[24] Viswanathan V Kumpatla S Aravindalochanan V Rajan R Chinnasamy C Srinivasan R. Prevalence of diabetes and pre-diabetes and associated risk factors among tuberculosis patients in India. PLoS ONE. (2012) 7:e41367. 10.1371/journal.pone.0041367 22848473 PMC3406054

[25] Wang Q Ma A Han X Zhao S Cai J Ma Y. Prevalence of type 2 diabetes among newly detected pulmonary tuberculosis patients in China: a community based cohort study. PLoS ONE. (2013) 8:e82660. 10.1371/journal.pone.0082660 24367535 PMC3867381

[26] Amare H Gelaw A Anagaw B Gelaw B. Smear positive pulmonary tuberculosis among diabetic patients at the Dessie referral hospital, Northeast Ethiopia. Infect Dis Poverty. (2013) 2:6. 10.1186/2049-9957-2-6 24499664 PMC3707095

[27] Workneh MH Bjune GA Yimer SA. Prevalence and associated factors of diabetes mellitus among tuberculosis patients in south-eastern amhara region, ethiopia: a cross sectional study. PLoS ONE. (2016) 11:e0147621. 10.1371/journal.pone.0147621 26808967 PMC4726615

[28] Shidam UG Roy G Sahu SK Kumar SV Ananthanarayanan PH. Screening for diabetes among presumptive tuberculosis patients at a tertiary care centre in Pondicherry, India. Int J Tuberc Lung Dis. (2015) 19:1163–8. 10.5588/ijtld.14.0971 26459527

[29] Lin YH Chen CP Chen PY Huang JC Ho C Weng HH. Screening for pulmonary tuberculosis in type 2 diabetes elderly: a cross-sectional study in a community hospital. BMC Public Health. (2015) 15:3. 10.1186/1471-2458-15-3 25572102 PMC4324855

[30] Ponnana M Sivangala R Joshi L Valluri V Gaddam S. IL-6 and IL-18 cytokine gene variants of pulmonary tuberculosis patients with co-morbid diabetes mellitus and their household contacts in Hyderabad. Gene. (2017) 627:298–306. 10.1016/j.gene.2017.06.046 28652186

[31] Huber FG Kristensen KL Holden IK Andersen PH Bakir B Jorgensen A. The prevalence of diabetes among tuberculosis patients in Denmark. BMC Infect Dis. (2022) 22:64. 10.1186/s12879-022-07048-4 35045811 PMC8767681

[32] Almeida-Junior JL Gil-Santana L Oliveira CA Castro S Cafezeiro AS Daltro C. Glucose metabolism disorder is associated with pulmonary tuberculosis in individuals with respiratory symptoms from Brazil. PLoS ONE. (2016) 11:e0153590. 10.1371/journal.pone.0153590 27078026 PMC4831681

[33] Awad SF Huangfu P Ayoub HH Pearson F Dargham SR Critchley JA. Forecasting the impact of diabetes mellitus on tuberculosis disease incidence and mortality in India. J Glob Health. (2019) 9:20415. 10.7189/jogh.09.020415 31673336 PMC6815875

[34] Gil-Santana L Almeida-Junior JL Oliveira CA Hickson LS Daltro C Castro S. Diabetes is associated with worse clinical presentation in tuberculosis patients from Brazil: a retrospective cohort study. PLoS ONE. (2016) 11:e0146876. 10.1371/journal.pone.0146876 26752596 PMC4709051

[35] Chiang CY Lee JJ Chien ST Enarson DA Chang YC Chen YT. Glycemic control and radiographic manifestations of tuberculosis in diabetic patients. PLoS ONE. (2014) 9:e93397. 10.1371/journal.pone.0093397 24699457 PMC3974751

[36] Arriaga MB Araujo-Pereira M Barreto-Duarte B Sales C Miguez-Pinto JP Nogueira EB. Prevalence and clinical profiling of dysglycemia and HIV infection in persons with pulmonary tuberculosis in Brazil. Front Med. (2021) 8:804173. 10.3389/fmed.2021.804173 35127760 PMC8814308

[37] Wu Z Guo J Huang Y Cai E Zhang X Pan Q. Diabetes mellitus in patients with pulmonary tuberculosis in an aging population in Shanghai, China: prevalence, clinical characteristics and outcomes. J Diabetes Complic. (2016) 30:237–41. 10.1016/j.jdiacomp.2015.11.014 26684166

[38] Chiang CY Bai KJ Lin HH Chien ST Lee JJ Enarson DA. The influence of diabetes, glycemic control, and diabetes-related comorbidities on pulmonary tuberculosis. PLoS ONE. (2015) 10:e0121698. 10.1371/journal.pone.0121698 25822974 PMC4378948

[39] Faurholt-Jepsen D Aabye MG Jensen AV Range N Praygod G Jeremiah K. Diabetes is associated with lower tuberculosis antigen-specific interferon gamma release in Tanzanian tuberculosis patients and non-tuberculosis controls. Scand J Infect Dis. (2014) 46:384–91. 10.3109/00365548.2014.885657 24621055

[40] Liu Q Li W Xue M Chen Y Du X Wang C. Diabetes mellitus and the risk of multidrug resistant tuberculosis: a meta-analysis. Sci Rep. (2017) 7:1090. 10.1038/s41598-017-01213-5 28439071 PMC5430797

[41] Rehman AU Khattak M Mushtaq U Latif M Ahmad I Rasool MF. The impact of diabetes mellitus on the emergence of multi-drug resistant tuberculosis and treatment failure in TB-diabetes comorbid patients: a systematic review and meta-analysis. Front Public Health. (2023) 11:1244450. 10.3389/fpubh.2023.1244450 38074769 PMC10704033

[42] Baker MA Harries AD Jeon CY Hart JE Kapur A Lonnroth K. The impact of diabetes on tuberculosis treatment outcomes: a systematic review. BMC Med. (2011) 9:81. 10.1186/1741-7015-9-81 21722362 PMC3155828

[43] Bezerra AL Moreira A Isidoro-Goncalves L Lara C Amorim G Silva EC. Clinical, laboratory, and radiographic aspects of patients with pulmonary tuberculosis and dysglycemia and tuberculosis treatment outcomes. J Bras Pneumol. (2022) 48:e20210505. 10.36416/1806-3756/e20210505 36449815 PMC9747138

[44] Viswanathan V Vigneswari A Selvan K Satyavani K Rajeswari R Kapur A. Effect of diabetes on treatment outcome of smear-positive pulmonary tuberculosis–a report from South India. J Diabetes Complications. (2014) 28:162–5. 10.1016/j.jdiacomp.2013.12.003 24461545

[45] Wang CS Yang CJ Chen HC Chuang SH Chong IW Hwang JJ. Impact of type 2 diabetes on manifestations and treatment outcome of pulmonary tuberculosis. Epidemiol Infect. (2009) 137:203–10. 10.1017/S0950268808000782 18559125

[46] Chiang CY Lee JJ Yu MC Enarson DA Lin TP Luh KT. Tuberculosis outcomes in Taipei: factors associated with treatment interruption for 2 months and death. Int J Tuberc Lung Dis. (2009) 13:105–11. 19105887

[47] Huangfu P Ugarte-Gil C Golub J Pearson F Critchley J. The effects of diabetes on tuberculosis treatment outcomes: an updated systematic review and meta-analysis. Int J Tuberc Lung Dis. (2019) 23:783–96. 10.5588/ijtld.18.0433 31439109

[48] Singla R Khan N Al-Sharif N Ai-Sayegh MO Shaikh MA Osman MM. Influence of diabetes on manifestations and treatment outcome of pulmonary TB patients. Int J Tuberc Lung Dis. (2006) 10:74–9. 16466041

[49] Singla R Raghu B Gupta A Caminero JA Sethi P Tayal D. Risk factors for early mortality in patients with pulmonary tuberculosis admitted to the emergency room. Pulmonology. (2021) 27:35–42. 10.1016/j.pulmoe.2020.02.002 32127307

[50] Arriaga MB Amorim G Queiroz ATL Rodrigues MMS Araujo-Pereira M Nogueira BMF. Novel stepwise approach to assess representativeness of a large multicenter observational cohort of tuberculosis patients: the example of RePORT Brazil. Int J Infect Dis. (2021) 103:110–8. 10.1016/j.ijid.2020.11.140 33197582 PMC7959330

[51] Xu G Hu X Lian Y Li X. Diabetes mellitus affects the treatment outcomes of drug-resistant tuberculosis: a systematic review and meta-analysis. BMC Infect Dis. (2023) 23:813. 10.1186/s12879-023-08765-0 37986146 PMC10662654

[52] Arriaga MB Rocha MS Nogueira BMF Nascimento V Araujo-Pereira M Souza AB. The effect of diabetes and prediabetes on *Mycobacterium tuberculosis* transmission to close contacts. J Infect Dis. (2021) 224:2064–72. 10.1093/infdis/jiab264 34008010 PMC8672762

[53] Riza AL Pearson F Ugarte-Gil C Alisjahbana B van de Vijver S Panduru NM. Clinical management of concurrent diabetes and tuberculosis and the implications for patient services. Lancet Diabetes Endocrinol. (2014) 2:740–53. 10.1016/S2213-8587(14)70110-X 25194887 PMC4852378

[54] Cho SK Yoon JS Lee MG Lee DH Lim LA Park K. Rifampin enhances the glucose-lowering effect of metformin and increases OCT1 mRNA levels in healthy participants. Clin Pharmacol Ther. (2011) 89:416–21. 10.1038/clpt.2010.266 21270793

[55] Te Brake LHM Yunivita V Livia R Soetedjo N van Ewijk-Beneken Kolmer E Koenderink JB. Rifampicin alters metformin plasma exposure but not blood glucose levels in diabetic tuberculosis patients. Clin Pharmacol Ther. (2019) 105:730–7. 10.1002/cpt.1232 30222857 PMC6587702

[56] Scheen AJ Paquot N. Metformin revisited: a critical review of the benefit-risk balance in at-risk patients with type 2 diabetes. Diabetes Metab. (2013) 39:179–90. 10.1016/j.diabet.2013.02.006 23528671

[57] Martinez N Ketheesan N West K Vallerskog T Kornfeld H. Impaired recognition of mycobacterium tuberculosis by alveolar macrophages from diabetic mice. J Infect Dis. (2016) 214:1629–37. 10.1093/infdis/jiw436 27630197 PMC5144731

[58] Martinez N Kornfeld H. Diabetes and immunity to tuberculosis. Eur J Immunol. (2014) 44:617–26. 10.1002/eji.201344301 24448841 PMC4213860

[59] Ayelign B Negash M Genetu M Wondmagegn T Shibabaw T. Immunological impacts of diabetes on the susceptibility of *Mycobacterium tuberculosis*. J Immunol Res. (2019) 2019:6196532. 10.1155/2019/6196532 31583258 PMC6754884

[60] Alba-Loureiro TC Hirabara SM Mendonca JR Curi R Pithon-Curi TC. Diabetes causes marked changes in function and metabolism of rat neutrophils. J Endocrinol. (2006) 188:295–303. 10.1677/joe.1.06438 16461555

[61] Lecube A Pachon G Petriz J Hernandez C Simo R. Phagocytic activity is impaired in type 2 diabetes mellitus and increases after metabolic improvement. PLoS ONE. (2011) 6:e23366. 10.1371/journal.pone.0023366 21876749 PMC3158070

[62] Pavlou S Lindsay J Ingram R Xu H Chen M. Sustained high glucose exposure sensitizes macrophage responses to cytokine stimuli but reduces their phagocytic activity. BMC Immunol. (2018) 19:24. 10.1186/s12865-018-0261-0 29996768 PMC6042333

[63] Wei R Li P Xue Y Liu Y Gong W Zhao W. Impact of diabetes mellitus on the immunity of tuberculosis patients: a retrospective, cross-sectional study. Risk Manag Healthc Policy. (2022) 15:611–27. 10.2147/RMHP.S354377 35431587 PMC9005360

[64] Martens GW Arikan MC Lee J Ren F Greiner D Kornfeld H. Tuberculosis susceptibility of diabetic mice. Am J Respir Cell Mol Biol. (2007) 37:518–24. 10.1165/rcmb.2006-0478OC 17585110 PMC2048677

[65] Vallerskog T Martens GW Kornfeld H. Diabetic mice display a delayed adaptive immune response to *Mycobacterium tuberculosis*. J Immunol. (2010) 184:6275–82. 10.4049/jimmunol.1000304 20421645 PMC2874741

[66] Kathamuthu GR Kumar NP Moideen K Menon PA Babu S. Decreased frequencies of gamma/delta T cells expressing Th1/Th17 cytokine, cytotoxic, and immune markers in latent tuberculosis-diabetes/pre-diabetes comorbidity. Front Cell Infect Microbiol. (2021) 11:756854. 10.3389/fcimb.2021.756854 34765568 PMC8577793

[67] Chen S Zhang W Zheng Z Shao X Yang P Yang X. Unraveling genetic causality between type 2 diabetes and pulmonary tuberculosis on the basis of Mendelian randomization. Diabetol Metab Syndr. (2023) 15:228. 10.1186/s13098-023-01213-8 37950319 PMC10636918

[68] Jiang Y Zhang W Wei M Yin D Tang Y Jia W. Associations between type 1 diabetes and pulmonary tuberculosis: a bidirectional mendelian randomization study. Diabetol Metab Syndr. (2024) 16:60. 10.1186/s13098-024-01296-x 38443967 PMC10913601

[69] Queiroz ATL Vinhaes CL Fukutani ER Gupte AN Kumar NP Fukutani KF. A multi-center, prospective cohort study of whole blood gene expression in the tuberculosis-diabetes interaction. Sci Rep. (2023) 13:7769. 10.1038/s41598-023-34847-9 37173394 PMC10180618

[70] Eckold C Kumar V Weiner J Alisjahbana B Riza AL Ronacher K. Impact of intermediate hyperglycemia and diabetes on immune dysfunction in tuberculosis. Clin Infect Dis. (2021) 72:69–78. 10.1093/cid/ciaa751 32533832 PMC7823074

[71] Berry MP Graham CM McNab FW Xu Z Bloch SA Oni T. An interferon-inducible neutrophil-driven blood transcriptional signature in human tuberculosis. Nature. (2010) 466:973–7. 10.1038/nature09247 20725040 PMC3492754

[72] Cliff JM Lee JS Constantinou N Cho JE Clark TG Ronacher K. Distinct phases of blood gene expression pattern through tuberculosis treatment reflect modulation of the humoral immune response. J Infect Dis. (2013) 207:18–29. 10.1093/infdis/jis499 22872737

[73] Maertzdorf J Weiner J Mollenkopf HJ Network TB Bauer T Prasse A. Common patterns and disease-related signatures in tuberculosis and sarcoidosis. Proc Natl Acad Sci U S A. (2012) 109:7853–8. 10.1073/pnas.1121072109 22547807 PMC3356621

[74] Ottenhoff TH Dass RH Yang N Zhang MM Wong HE Sahiratmadja E. Genome-wide expression profiling identifies type 1 interferon response pathways in active tuberculosis. PLoS ONE. (2012) 7:e45839. 10.1371/journal.pone.0045839 23029268 PMC3448682

[75] Mayer-Barber KD Andrade BB Oland SD Amaral EP Barber DL Gonzales J. Host-directed therapy of tuberculosis based on interleukin-1 and type I interferon crosstalk. Nature. (2014) 511:99–103. 10.1038/nature13489 24990750 PMC4809146

[76] Liu T Wang Y Gui J Fu Y Ye C Hong X. Transcriptome analysis of the impact of diabetes as a comorbidity on tuberculosis. Medicine. (2022) 101:e31652. 10.1097/MD.0000000000031652 36596076 PMC9803411

[77] Rocha EF Vinhaes CL Araujo-Pereira M Mota TF Gupte AN Kumar NP. The sound of silent RNA in tuberculosis and the lncRNA role on infection. iScience. (2024) 27:108662. 10.1016/j.isci.2023.108662 38205253 PMC10777062

[78] DeFronzo RA Ferrannini E Groop L Henry RR Herman WH Holst JJ. Type 2 diabetes mellitus. Nat Rev Dis Primers. (2015) 1:15019. 10.1038/nrdp.2015.19 27189025

[79] Arriaga MB Araujo-Pereira M Barreto-Duarte B Nogueira B Freire M Queiroz ATL. The effect of diabetes and prediabetes on antituberculosis treatment outcomes: a multicenter prospective cohort study. J Infect Dis. (2022) 225:617–26. 10.1093/infdis/jiab427 34651642 PMC8844586

[80] Pavan Kumar N Moideen K Nancy A Viswanathan V Shruthi BS Shanmugam S. Plasma eicosanoid levels in tuberculosis and tuberculosis-diabetes co-morbidity are associated with lung pathology and bacterial burden. Front Cell Infect Microbiol. (2019) 9:335. 10.3389/fcimb.2019.00335 31632923 PMC6779700

[81] Prada-Medina CA Fukutani KF Pavan Kumar N Gil-Santana L Babu S Lichtenstein F. Systems immunology of diabetes-tuberculosis comorbidity reveals signatures of disease complications. Sci Rep. (2017) 7:1999. 10.1038/s41598-017-01767-4 28515464 PMC5435727

[82] He X Wang Y Yang Y He Q Sun L Jin J. Quantitative proteomics reveals plasma protein profile and potential pathways in pulmonary tuberculosis patients with and without diabetes. Tuberculosis. (2023) 143:102424. 10.1016/j.tube.2023.102424 37871493

[83] Kundu J Bakshi S Joshi H Bhadada SK Verma I Sharma S. Proteomic profiling of peripheral blood mononuclear cells isolated from patients with tuberculosis and diabetes copathogenesis - A pilot study. PLoS ONE. (2020) 15:e0233326. 10.1371/journal.pone.0233326 33156824 PMC7647457

[84] Chaudhary S Pahwa F Nanda RK. Dysregulated cysteine metabolism leads to worsened liver pathology in diabetes-tuberculosis comorbid condition. J Biol Chem. (2024) 300:105634. 10.1016/j.jbc.2024.105634 38199571 PMC10850780

[85] Amaral EP Vinhaes CL Oliveira-de-Souza D Nogueira B Akrami KM Andrade BB. The interplay between systemic inflammation, oxidative stress, and tissue remodeling in tuberculosis. Antioxid Redox Signal. (2021) 34:471–85. 10.1089/ars.2020.8124 32559410 PMC8020551

[86] Kumar NP Fukutani KF Shruthi BS Alves T Silveira-Mattos PS Rocha MS. Persistent inflammation during anti-tuberculosis treatment with diabetes comorbidity. Elife. (2019) 8:e46477. 10.7554/eLife.46477 31271354 PMC6660216

[87] Yen NTH Anh NK Jayanti RP Phat NK Vu DH Ghim JL. Multimodal plasma metabolomics and lipidomics in elucidating metabolic perturbations in tuberculosis patients with concurrent type 2 diabetes. Biochimie. (2023) 211:153–63. 10.1016/j.biochi.2023.04.009 37062470

[88] Lopez-Hernandez Y Lara-Ramirez EE Salgado-Bustamante M Lopez JA Oropeza-Valdez JJ Jaime-Sanchez E. Glycerophospholipid metabolism alterations in patients with type 2 diabetes mellitus and tuberculosis comorbidity. Arch Med Res. (2019) 50:71–8. 10.1016/j.arcmed.2019.05.006 31349956

[89] Arriaga MB Karim F Queiroz ATL Araujo-Pereira M Barreto-Duarte B Sales C. Effect of dysglycemia on urinary lipid mediator profiles in persons with pulmonary tuberculosis. Front Immunol. (2022) 13:919802. 10.3389/fimmu.2022.919802 35874781 PMC9304990

[90] Vrieling F Alisjahbana B Sahiratmadja E van Crevel R Harms AC Hankemeier T. Plasma metabolomics in tuberculosis patients with and without concurrent type 2 diabetes at diagnosis and during antibiotic treatment. Sci Rep. (2019) 9:18669. 10.1038/s41598-019-54983-5 31822686 PMC6904442

[91] Vinhaes CL Oliveira-de-Souza D Silveira-Mattos PS Nogueira B Shi R Wei W. Changes in inflammatory protein and lipid mediator profiles persist after antitubercular treatment of pulmonary and extrapulmonary tuberculosis: a prospective cohort study. Cytokine. (2019) 123:154759. 10.1016/j.cyto.2019.154759 31226436 PMC6739167

[92] Vinhaes CL Fukutani ER Santana GC Arriaga MB Barreto-Duarte B Araujo-Pereira M. An integrative multi-omics approach to characterize interactions between tuberculosis and diabetes mellitus. iScience. (2024) 27:109135. 10.1016/j.isci.2024.109135 38380250 PMC10877940

